# Reduced Order Multiscale Simulation of Diffuse Damage in Concrete

**DOI:** 10.3390/ma14143830

**Published:** 2021-07-08

**Authors:** Giao Vu, Fabian Diewald, Jithender J. Timothy, Christoph Gehlen, Günther Meschke

**Affiliations:** 1 Institute for Structural Mechanics, Ruhr University Bochum, Universitaetsstrasse 150, 44801 Bochum, Germany; thi.vu-h6d@rub.de (G.V.); guenther.meschke@rub.de (G.M.); 2 Chair of Materials Science and Testing, Centre for Building Materials, Technical University of Munich, Franz-Langinger-Strasse 10, 81245 Munich, Germany; fabian.diewald@tum.de (F.D.); gehlen@tum.de (C.G.)

**Keywords:** concrete, mesoscale, reduced order multiscale simulation, microcracking, micromechanics, linear elastic fracture mechanics, anisotropic damage

## Abstract

Damage in concrete structures initiates as the growth of diffuse microcracks that is followed by damage localisation and eventually leads to structural failure. Weak changes such as diffuse microcracking processes are failure precursors. Identification and characterisation of these failure precursors at an early stage of concrete degradation and application of suitable precautionary measures will considerably reduce the costs of repair and maintenance. To this end, a reduced order multiscale model for simulating microcracking-induced damage in concrete at the mesoscale level is proposed. The model simulates the propagation of microcracks in concrete using a two-scale computational methodology. First, a realistic concrete specimen that explicitly resolves the coarse aggregates in a mortar matrix was generated at the mesoscale. Microcrack growth in the mortar matrix is modelled using a synthesis of continuum micromechanics and fracture mechanics. Model order reduction of the two-scale model is achieved using a clustering technique. Model predictions are calibrated and validated using uniaxial compression tests performed in the laboratory.

## 1. Introduction

Concrete is a heterogeneous, multiphase material with a disordered material structure across multiple length scales. At the mesoscopic scale ($10^{-2}$ m $<l<10^{-1}$ m), concrete is characterised by coarse aggregates of various shapes and sizes embedded in a cementitious mortar material. The morphology of the mortar matrix around the length scale of $10^{-3}$ m is also highly heterogeneous and comprises of the hardened cement paste and the fine aggregates (sand). At length scales smaller than $10^{-4}$ m, cement paste is characterised by a C-S-H matrix hosting clinker phases, the CH crystal, and capillary porosity [1]. It is well known that cementitious materials such as concrete, mortar, or cement paste contain initial microcracks and defects, typically distributed diffusively within the material, arising from autogeneous and drying shrinkage of the material. Subjected to external loadings, heterogeneities such as coarse aggregates and pores induce a highly disordered stress field within the material. Interaction of the initial stresses with the loading-induced evolution of initial defects and pre-existing microcracks results in a complex damage process on the mesostructure of the material [2,3]. Damage in concrete initiates at defects and pre/existing microcracks and propagates, leading to microcrack coalescence i.e., crack localisation, eventually leading to the complete loss of the load-bearing capacity of the concrete structure. Despite its infinitesimal size, pre-existing microcracks and their evolution significantly determine the behaviour of concrete subjected to external loadings. In this context, surface tomography analysis [4,5] can be used to provide additional information on the load induced microcracking behavior in concrete. Moreover, microcracking in concrete initiates already at a load levels much lower than the ultimate load [6,7,8,9,10,11]. Thus, from a structural health monitoring point of view, detection of microcracking, which is a precursor to complete failure of the structure, can help take suitable precautionary measures in advance [12,13]. Due to the high sensitivity of the so-called coda waves, Coda Wave Interferometry techniques (CWI) can be used to detect weak changes such as microcracking in concrete [14,15,16]. However, research in this direction is challenging and still remains at a very early stage far from practical application.

The synthesis of computational modelling and experimental techniques can significantly accelerate the development of a reliable methodology to detect microcracking induced damage in concrete. Having this goal in mind, this paper presents a computational model for simulating microcracking induced damage in the pre-peak regime of concrete, taking into account the heterogeneity of concrete at the mesoscale and, in particular, the role of the aggregates. It is to be noted that the term ‘diffuse damage’, also called diffuse microcracking or distributed microcracking in the paper, is associated with the stable propagation of preexisting microcracks caused by the mechanical load. This microcracking manifests itself at the macroscopic level as a gradual reduction in the stiffness of concrete in the pre-peak regime.

Several computational modelling strategies ranging from phenomenological macroscopic models, continuum micromechanics based models, mesoscale simulations to multiscale models have been proposed in the literature. Phenomenological damage and plasticity-damage models (e.g., [17,18,19,20,21,22,23], just to mention a few) consider concrete as a homogeneous medium. They are calibrated based on stress–strain relations from tensile and compression tests and are suitable for simulating damage and the ultimate load of large-scale concrete structures. However, no information on the microstructural changes during loading is included in this type of macroscopic model.

In contrast, continuum micromechanics models are able to approximately model the interactions of the heterogeneities (e.g., microcracks, aggregates, etc.) across multiple length scales using multi-level homogenisation schemes (see for e.g., [1,24,25]). Due to the mean-field assumption, damage within this framework is assumed to be represented by diffusely distributed flat or penny-shaped inclusions that are embedded in a continuous matrix ([26,27,28,29]). The evolution of the microcrack geometry is governed by fracture mechanics (e.g., [25,30]), or phenomenological damage laws [31,32]. As these models are analytical or semi-analytical formulations, they are computationally inexpensive. On the other hand, mesoscopic modelling approaches explicitly resolve the individual components of the material, see, e.g., [33,34,35,36,37,38,39,40,41,42]. Mesoscale models can be formulated using a variety of discretisation methods, such as the Finite Element Method (FEM) [33,34,39,43,44], the Discrete Element Method (DEM) [45], and Fast Fourier Transform (FFT)-based homogenisation methods [46,47,48]. Among these methods, the FFT homogenisation approach based on the Lippmann–Schwinger equation has recently gained in popularity for the analysis of materials with a complex morphology. This method allows a direct use of image-based data structures describing materials obtained from CT scans or other imaging techniques and it outperforms FEM and DEM in terms of computational efficiency and memory footprint.

Inevitably, the range of length scales that can be considered using computational mesoscale models is limited. Thus, for concrete, given the wide range of length scales involved, micromechanics-based multiscale modeling, in conjunction with model-order reduction techniques such as proper orthogonal decomposition [49,50,51], data-driven reduced-order PSP linkages [52], and the recently introduced clustering-based homogenisation methods [53,54,55], is essential. The self-consistent analysis (SCA) methods proposed in [53,54,55] offer high cost efficiency in terms of training data requirement and leads to a substantial reduction of the degrees of freedoms from a few million to only a few hundred.

### Goals and Structure of the Paper

The aim of the paper is to model the load induced distributed microcracking phenomenon in concrete by means of a multiscale reduced order modelling approach. To this end, we have developed a multiscale model, characterised by the combination of continuum micromechanics and fracture mechanics on the microcrack level and a direct computational resolution of the mesoscale of concrete to describe load induced microcrack evolution. We demonstrate the predictive capability of the model by validations with experimental measurements.

The remainder of the paper is organised as follows: In Section 2, we provide key results from the experimental program devoted to the validation of the proposed model. Section 3 addresses the model description at the meso- and microscopic scales. In Section 4, we present the k-means based model reduction procedure as well as a series of numerical experiments. Proceeding to Section 5, calibration and validation procedure of the proposed model is presented, and the results are discussed. Finally, in Section 6, we summarise the paper and provide concluding remarks.

## 2. Experimental Program

### 2.1. Material and Specimen Preparation

**Figure 1:**
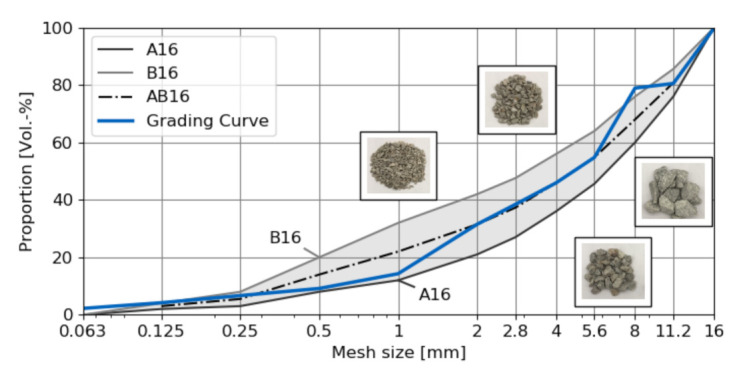
Standardised and optimised aggregate distributions for a concrete mix with an approximated AB16 aggregate distribution.

In order to support and validate the proposed model, three cubes (a = 10 cm) made of concrete and mortar serve as test specimens. We used ordinary Portland cement with a water-to-cement ratio of 0.45 for both types of specimens, crushed aggregates with an AB16 grading curve (Figure 1) and a cement content of 350 kg/m${}^{3}$ for the concrete (see Table 1 for a list of the raw materials in this concrete composition). The predominantly quartzitic aggregates used in the material composition come from the Taunus region in Germany and are available in four different grain sizes [0/2, 2/5.6, 5.6/8, 8/16] mm. The aggregates were nearly purely quartzitic which is favorable in order to minimize variations of mechanical properties due to variable minerals and their proportions. For the mortar specimens, we added quartz powder (φ=30.34%) with an average grain size of $d_{50\%}$ = 8 μm and an upper grain size of $d_{95\%}$ = 25 μm to increase resistance against shrinkage. Shrinkage-induced cracks should be prevented as specimens were tested after more than one year to ensure almost complete hydration. The specimens have been cured with the following conditioning procedure: After the production day (t = 0), specimens harden for one day. Thereafter, the specimens were conditioned under water for 56 days at T = 20 ${}^{\circ }$C. Then, the specimens were conditioned at T = 20 ${}^{\circ }$C and RH = 65% until the test. It is to be noted that no considerable difference to be expected between the strength and mechanical properties of specimens tested after one month and after one year. In this paper, we use the terms grains and aggregates interchangeably.

### 2.2. Characterisation of the Mesostructure of Concrete

The volume fractions of aggregates and air pores, along with their spatial distribution, are key parameters for characterising the mesostructure of concrete specimens and serve as input data for the generation of synthetic concrete mesostructure models. For the test specimens, an optimised aggregate composition was computed using the Generalised Reduced Gradient Method [56] by minimisation of the residuals between the cumulative proportions of four aggregate fractions at the specified discrete points (Figure 1) and the ideal AB16 aggregate composition according to building standards [57,58].

A more refined quantification procedure of the quartzitic aggregates was also carried out to extract the statistical data required for the generation of synthetic concrete mesostructures. The resulting absolute volume fraction of each concrete constituent is listed in Table 2. As can be seen, we further classify the aggregates into two sub-categories according to their size: fine and coarse aggregates. Here, the ’threshold’ value is set to 3 mm. Thus, the volume fraction of cement paste matrix, fine aggregates, and coarse aggregates are 29.259%, 22.448%, and 48.292%, respectively.

### 2.3. Determination of Elastic Properties of Concrete and Its Constituents

The proposed multiscale model also requires data regarding the mechanical properties of the material composition. Thus, a series of tests were performed to determine the properties of mortar, quartzitic aggregates and concrete of standard AB16. The testing machine is a Walter+Bai AG DB 3000/300 kN with a digital controller, Digicon 2000 (Löhningen, Switzerland). In order to design the testing procedure, we used the materials testing software “Proteus”. The material parameters of interest include the Young’s modulus, Poisson’s ratio, and the compressive strength. These parameters are summarised for each constituent in Table 3.

**Figure 2:**
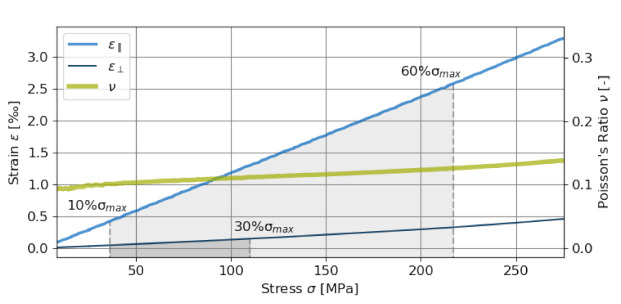
Stress–strain diagrams for longitudinal strain $\varepsilon_{\|}$ and lateral strain $\varepsilon_{⊥}$ obtained from uniaxial compression tests on a cylindrical quartzitic specimen. The strain gauges failed before reaching the ultimate compressive strength of 368.0 MPa. The plot also shows the Poisson’s ratio obtained from the ratio of the lateral and the longitudinal strains.

To measure the Young’s Modulus and the Poisson’s ratio of the quartzitic aggregates, a uniaxial compression test is performed on a quartzitic sample of cylindrical geometry. The cylinder with 5 cm diameter and 10 cm height was extracted from the same quartzitic material as the concrete aggregates. The specimen is subjected to a load controlled test and two strain gauges, arranged in a diagonal bridge circuit, were used to monitor the longitudinal and lateral strains on the aggregate surface [59,60]. Anderson–Darling-Tests [61] show a linear relationship up to 60% of the ultimate compressive strength $\sigma_{max}$ for longitudinal strain and up to 30% $\sigma_{max}$ for lateral strain, assuming a normal residual distribution between the experimental values and a linear regression function using a *p*-value of 5% (see Figure 2). The derived parameters for the quartzitic aggregates are in line with data from the literature [62], whereas the compressive strength is slightly smaller (90 GPa) and Poisson’s ratio slightly larger (0.10) as compared to α-Quartz.

**Figure 3:**
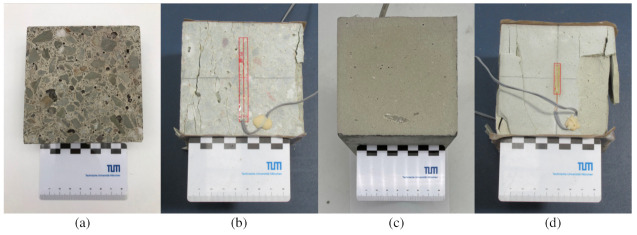
Determination of material parameters for concrete and cement paste: (**a**) polished concrete specimen with a maximum grain size of 16 mm before loading, (**b**) cracked concrete specimen with a strain gauge after the compressive load test, (**c**) polished hardened cement paste specimen before loading, (**d**) hardened cement paste specimen with strain gauge after the compressive load test.

The Young’s modulus for concrete and mortar samples is also obtained from a uniaxial compression test. Three samples of size 10 cm${}^{3}$ were loaded in a displacement controlled test with a displacement rate 0.1 mm/h. To accurately measure the true longitudinal deformation of the specimens, two external strain gauges (DD1 displacement transducer) were used. The Young’s modulus was estimated and averaged using a linear regression between two points from the stress–strain curve, at 10% and 30% of the maximum compressive stress. In all tests, Polytetrafluoroethylene (PTFE) films were placed between the samples and the loading platens to reduce friction. Figure 3 shows the specimens in a sound state and after displacement controlled compressive loading tests up to the ultimate state.

## 3. Scale-Bridging Modelling of Cementitious Materials

### 3.1. The Scale-Bridging Modelling Concept

When subjected to external mechanical loads, damage in concrete initiates from pre-existing defects and microcracks. These microcracks grow and ultimately coalesce to form visible macroscopic localised cracks. The growth of microcracks in concrete is completely governed by the presence and distribution of the heterogeneities (aggregates, pores, defects, initial microcracks, etc.). Moreover, it involves mechanisms that interact across multiple length scales i.e., the loading is applied at the macroscopic scale, the coarse aggregates serve as stress concentrations at the mesoscopic scale and the microcracks initiate and start growing at the microscopic scale. In order to bridge these scales, a multiscale model that takes into account the most essential physics contributing to the damage evolution in concrete is required.

**Figure 4:**
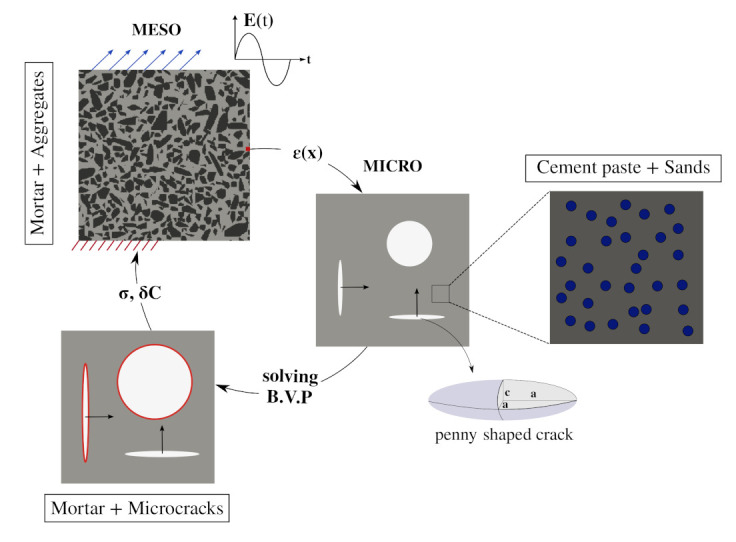
Schematic illustration of the multiscale computational homogenisation approach considered in this paper.

In this paper, a reduced order model for scale-bridging modelling of damage evolution in concrete is formulated. Figure 4 illustrates the proposed modelling procedure. First, we consider a representative elementary volume (REV) at the mesoscale. At this scale, the coarse aggregates are explicitly resolved. At each mesoscopic point, an associated microscopic representative elementary volume (REV) is incorporated. Hence, the REV at the mesoscale bridges the applied macroscopic loading at the macroscale and the growth of microcracks at the microscale. At the microscopic scale, the mortar solid consists of an intact mortar matrix and pre-existing microcracks as weak inclusions. The mortar solid is idealised as a multi-phase material with spherical fine aggregates embedded in the cementitious matrix. Microcracks are modelled using three-sets of mutually orthogonal penny-shaped microcrack families, see Section 3.2. At the mesoscale, concrete is explicitly represented in a computational model as a two-phase composite consisting of a mortar matrix and coarse aggregates. The aggregates are assumed to be linear elastic, while the nonlinear behaviour of the mortar matrix is modelled at the microscopic scale using a combination of continuum micromechanics and Linear Elastic Fracture Mechanics [25].

In summary, this modelling approach entails separate model descriptions at different scales as well as the coupling relations between the scales. The interaction among scales is realised through homogenisation and localisation procedures. In the homogenisation process, information at lower scales is transferred to the higher scale through physically consistent averaged quantities (e.g., macroscopic stresses, effective stiffness). Localisation (also called concentration in the literature) is a downscaling procedure that relates strain or stress measures across the scales.

See Appendix A for the algorithmic implementation.

### 3.2. Microscale Model: Microcracking in the Mortar Material

#### 3.2.1. Model Description

In order to model distributed microcracking in the mortar material, we adopt the multiscale concrete model proposed in [25]. The model is based on the synthesis of continuum micromechanics and Linear Elastic Fracture Mechanics (LEFM) (see [30]). At the microscale, a mortar REV of size *l* is considered, which is consisting of a mortar solid matrix and microcracks as inclusions. Microcracks represent the initial defects in the mortar and their evolution accounts for the fracture and damage mechanism at this length scale.

Three microcrack families, embedded in an "intact" mortar matrix material and oriented in three mutually orthogonal planes aligned with the major axes, are considered as inclusions. The geometry of these microcracks is idealised as penny-shape (oblate ellipsoid) with aspect ratio $X=\frac{a}{c}\gg 1$, microcrack radius *a*, and half microcrack opening *c*. The volume fraction of each microcrack family is evaluated as $\varphi_{cr,i}=\frac{4}{3}\pi N_{i}X_{i}a_{i}^{3},\left(i=1,2,3\right)$, where $N_{i}$ is the number of microcracks per unit volume. The dimensionless *crack density parameter*$\gamma_{i}=N_{i}a_{i}^{3}.$ Given the microcrack volume fractions and the elastic properties of the mortar matrix $C_{m}$, the initial effective stiffness $C_{0}^{eff}$ of mortar REV can be estimated using analytical homogenisation schemes (for e.g., Mori–Tanaka scheme used in [25,30]).

#### 3.2.2. Microcrack Growth

When concrete is subjected to loading, the growth of microcracks is idealised in the model as an extension of microcrack radius. Microcracks may grow only if they satisfy the criterion:

(1) $$-\frac{1}{2}\tilde{E}:\frac{\partial C^{eff}}{\partial \gamma_{i}}:\tilde{E}\leq \frac{2\pi }{3}\frac{g_{f}}{a_{i}},\;\left(i=1,2,3\right),$$

where E is the applied strain obtained from the mesoscale simulation. $\gamma_{i}$ is the dimensionless crack density parameter and $g_{f}$ is the microscopic fracture energy. $\tilde{E}$ denotes the equivalent strain, which is defined as the positive part of E [63]. The homogenised stiffness $C^{eff}$ is a function of $C_{m},a_{i},X_{i}$, and $N_{i}$.

Equation (1) involves solving a system of three coupled inequalities for the current microcrack radius $a_{i}$. The partial derivative of $C^{eff}$ with respect to the microcrack density parameter $\gamma_{i}$ is evaluated accurately using the complex-step derivative. The computation of $a_{i}$ at a given load state is computed iteratively using the Newton–Raphson algorithm as follows. Let $r_{i}\left(a\right)$ be the residual

(2) $$\begin{array}{cc} r_{i}\left(a\right) & =-\frac{1}{2}\tilde{E}:\frac{\partial C^{eff}}{\partial \gamma_{i}}:\tilde{E}-\frac{2\pi }{3}\frac{g_{f}}{a_{i}}, \end{array}$$

and $a={\left(a_{1},a_{2},a_{3}\right)}^{T}$ denotes the microcrack-radii, then $g_{ij}$ expresses the partial derivative of $r_{i}$ with respect to $a_{j}$. This derivative is approximated using the numerical derivative technique

(3) $$\begin{array}{cc} g_{ij} & =\nabla_{a}r=r_{i,j},\;\left(i,j=1,2,3\right). \end{array}$$

At iteration *k*, the microcrack radius increment is computed as

(4) $$\begin{array}{c} \Delta a_{i}=a_{i}^{k}-g_{ij}r_{j}, \end{array}$$

and the microcrack radius is updated accordingly as

(5) $$\begin{array}{c} a_{i}^{k+1}=a_{i}^{k}+\Delta a_{i}. \end{array}$$

With tol being a small value close to zero (e.g., $10^{-6}$), the convergence criterion is expressed as

(6) $$\begin{array}{c} r_{i}\leq tol. \end{array}$$

After a converged microcrack radius is computed, the reduced effective stiffness tensor corresponding to the current microcrack configuration is updated. It should be noted that each microcrack family is allowed to grow independently, and, therefore, depending on the direction of microcrack growth, an anisotropy of the homogenised stiffness is induced.

#### 3.2.3. Analysis of a Micro-Cracked Mortar REV under Uniaxial Loading Tests Using the Mori–Tanaka Homogenisation Scheme

In order to verify the proposed microcracking model, we simulate a uniaxial tension and uniaxial compression loading on a mortar REV. The investigated mortar REV consists of fine aggregates (sand) with a volume fraction of 35% embedded in a cement matrix. The material stiffness for the cement matrix and the aggregates are 21.6 GPa (taken from [24]) and 84.6 GPa, respectively. The Poisson ratio of sand inclusions is assumed to be 0.2 [25]. The model parameters including the geometrical parameters are chosen within the range proposed in [25].

A summary of input and calibrated parameters as well as the numerical results can be found in Table 4. It is to be noted that $E_{s}$and $\nu_{s}$ correspond to the elasticity parameters of a theoretical cement paste without microcracks. These values are calibrated to obtain the effective Young’s modulus of hardened cement paste of 21.6 GPa and Poisson ratio 0.15.

**Figure 5:**
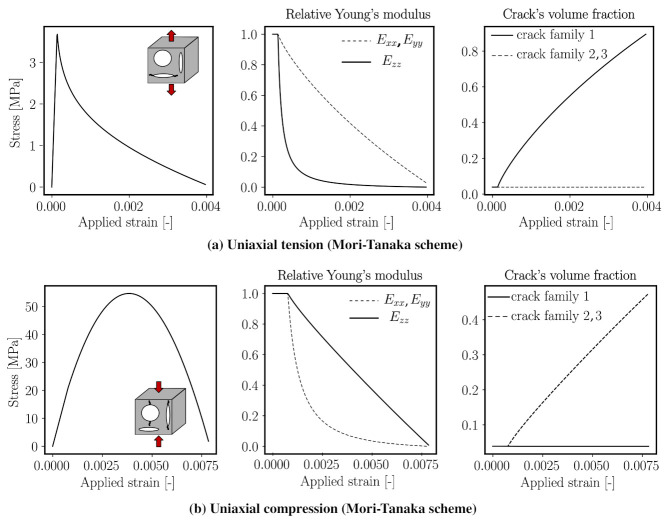
(Left) stress–strain relation of mortar subjected to uniaxial tension (top) and uniaxial compression (bottom). (Center) stiffness evolution due to the propagation of microcracks. (Right) growth of the volume fraction of three microcrack families. Microcrack propagation is modelled as the increase in microcrack radius according to the Griffith criterion.

In this numerical experiment, the micro-cracked mortar REV is assumed to contain an initial microcrack volume fraction of 11.79% with aspect ratio 17. As a consequence of introducing initial microcracks, the mortar REV stiffness reduces to 26.9 GPa at the zero-stress state. Figure 5 top and bottom left show the stress and strain responses of the mortar REV subjected to uniaxial tension and compression, respectively. Under uniaxial tension, microcrack family 1, which is oriented in a direction perpendicular to the maximum stress, propagates. This microcrack propagation results in a significant reduction of the stiffness in a longitudinal direction (*z*-direction in this particular numerical example) and a slight reduction in the stiffness in the transversal directions. The volume fraction of microcracks belonging to family 1 grows with increasing loads while the volume fraction of the other (two families) remains constant.

When subjected to uniaxial compression, microcrack families 2 and 3 grow due to the effect of the Poisson ratio. This leads to a dramatic reduction of material stiffnesses in the transversal directions, while $E_{zz}$ reduces at a much slower rate. For the given parameter set, under compression, microcracking initiates at a load level 38.1% of the compressive strength. Both the computed compressive and tensile strengths are within the standard range for mortar composite. It should be noted that the proposed micromechanics model is computationally cheap and provides an almost instant upscaling tool, in comparison with explicit computational modelling of discrete microcracks, and therefore is ideally suited to be combined with a computational model at a larger scale. In addition, depending on the direction of microcrack growth, anisotropic damage can be simulated, as this information is directly obtained from the anisotropic homogenised stiffness tensor. A detailed parametric study of the influence of the model parameters on various characteristics of the compression behaviour of mortar is presented in Appendix B for interested readers.

### 3.3. Computational Model of Concrete on the Mesoscopic Scale

At the mesoscopic scale, concrete is idealised numerically in terms of a two-phase composite occupying a given volume $\Omega_{M}$, where the subscript “M” denotes quantities at the mesoscale. The numerical concrete specimen exhibits a periodic microstructure and is discretised using a uniform three-dimensional grid of $N^{3}$ voxels. A concrete mesostructure generator (CMG) has been developed by authors [65] that allows an efficient computation of voxel-based synthetic concrete numerical samples. The algorithm is capable of generating realistic concrete periodic mesostructures given the aggregate size distribution. A Python implementation of the Concrete Mesostructure Generator (pyCMG, [65]) is also available.

The stress and strain fields σ(x),ε(x) of a concrete REV subjected to a prescribed macroscopic strain $E_{0}$ are computed by solving the integral form of the periodic Lippmann–Schwinger equation

(7) $$\begin{array}{c} \varepsilon \left(x\right)+\int_{\Omega_{M}}\Gamma^{0}\left(x-y\right):\left(C\left(x\right)-C^{0}\right):\varepsilon \left(y\right)dy=E_{0}, \end{array}$$

where $\Gamma^{0}$ denotes the periodic Green tensor of the reference elasticity tensor $C^{0}$. The explicit expression of the Green tensor for an isotropic elastic reference material with Lame parameters λ and μ in Fourier space is given as follows [46]:

(8) $$\begin{array}{cc} {\hat{\Gamma }}_{0} & =\frac{1}{{4\mu |\xi |}^{2}}\left(\delta_{ki}\xi_{l}\xi_{j}+\delta_{li}\xi_{k}\xi_{j}+\delta_{kj}\xi_{l}\xi_{i}+\delta_{lj}\xi_{k}\xi_{i}\right)-\frac{\lambda +\mu }{\mu (\lambda +2\mu )}\frac{\xi_{i}\xi_{j}\xi_{k}\xi_{l}}{{\left|\xi \right|}^{4}}, \end{array}$$

(9) $$\begin{array}{cc} \xi_{i} & =\frac{2\pi m_{i}}{N},\;m_{i}=-\left(N-1\right)/2,...,\left(N-1\right)/2, \end{array}$$

with ξ and $\hat{.}$ denoting the frequency vector in Fourier space and Fourier formulation of the respective field, respectively. Equation (7) can be conveniently reformulated such that it can be solved iteratively using fixed-point iteration [46] in Fourier space:

(10) $$\begin{array}{cc} {\hat{\varepsilon }}^{k+1}\left(\xi \right) & ={\hat{\varepsilon }}^{k}-{\hat{\Gamma }}^{0}\left(\xi \right):{\hat{\sigma }}^{k}\left(\xi \right)\;\;\forall \;\;\xi \neq 0, \end{array}$$

(11) $$\begin{array}{cc} \hat{\varepsilon }\left(0\right) & =E_{0}. \end{array}$$

For the iterative solution, strain-based convergence criterion

(12) $$\begin{array}{c} \eta =\frac{\left|\right|\varepsilon^{k+1}-\varepsilon^{k}\left|\right|}{\left|\right|E_{0}\left|\right|}<tol, \end{array}$$

is used.

## 4. Model Reduction Using K-Means Clustering

An explicit multiscale approach without using proper model reduction would result in an explosion of the number of unknowns, especially in the case of complex three-dimensional microstructures. To accelerate material analysis and design, one looks for a strategic way to reduce the computational complexity of the model, both in terms of computation time and data storage requirement. In our work, the clustering technique proposed by [53] is adopted. The method couples the data-driven approach using the k-means clustering algorithm to efficiently characterise the salient features of a microstructure, with the clustered FFT-based solver. Essentially, the methodology consists of two steps:

- *Offline stage* Pre-computation of a reduced-order dataset characterising the behaviour of a given REV by decomposing the entire domain of the high fidelity REV into a set of sub-domains (often denoted as clusters) and computing the so-called interaction tensors $D_{IJ}$ for all cluster pairs.
- *Online stage* Actual computation of the response of the REV for various loading conditions using the reduced-order dataset obtained from the *offline stage*.

### 4.1. K-Means Model Reduction Procedure

#### 4.1.1. Offline Stage

In the offline stage, the k-means clustering procedure was employed for grouping voxels that deform in a similar fashion. The algorithm requires a collection of data characterising the behaviour of all voxels under various loading conditions. To this end, fine-scale simulations of six orthogonal unit loading conditions were performed on the concrete REV and the corresponding strain fields are recorded, as suggested in [53]. In our implementation, training data in the offline stage were generated by means of the Lippmann–Schwinger based FFT solver described in Section 3.3. Alternatively, such calculation can also be performed using a commercial FEM software (e.g., [66,67]).

The metric used to group the voxels is the localisation tensor denoted as A(x), which basically maps the local elastic response of the material to the macroscopic strain E:

(13) $$\begin{array}{cc} \varepsilon (x) & =A(x):E. \end{array}$$

As a result, the k-means clustering algorithm groups the voxels into one cluster based on the similarity of their localisation tensors. Full details regarding the construction of A(x) and the k-means clustering algorithm can be found in [53].

Next, for the sake of clarity, but without going into too much detail, we shall provide the basic formulation of the interaction tensor $D_{IJ}$. $D_{IJ}$ is defined as

(14) $$\begin{array}{c} D_{IJ}=\frac{1}{c^{I}V}\int_{\Omega }\int_{\Omega }X^{I}\left(x\right)X^{J}\left(x^{\prime }\right)\Gamma^{0}\left(x,x^{\prime }\right)dx^{\prime }dx, \end{array}$$

with $c_{I}$ being the volume fraction of the $I^{th}$ cluster. $X^{I}$ is the characteristic function in the domain of the $I_{th}$ material cluster $\Omega^{I}$, defined as

(15) $$\begin{array}{c} X^{I}\left(x\right)=\left\{\begin{array}{cc} 1 & \forall x\in \Omega^{I}\\ 0 & \mathrm{otherwise}. \end{array}\right. \end{array}$$

#### 4.1.2. Online Stage

The equilibrium stress and strain for each cluster ($\varepsilon^{I},\;\sigma^{I}$) for a loading condition $E_{n}$ are computed using the cluster based Lippmann–Schwinger equation, re-formulated as

(16) $$\begin{array}{cc} \varepsilon_{n,I}+\sum_{J=1}^{nJ}D_{IJ}:\left(\sigma_{n,J}-C^{0}:\varepsilon_{n,J}\right) & =E_{n}, \end{array}$$

where n,J is the number of material clusters, and Equation (16) can be rearranged so that it can be solved using the fixed point iteration algorithm as follows:

(17) $$\begin{array}{cc} \varepsilon_{n,I}^{k+1} & =-\sum_{J=1}^{nJ}D_{IJ}:\left(\sigma_{n,J}^{k}-C^{0}:\varepsilon_{n,J}^{k}\right)+E_{n}, \end{array}$$

(18) $$\begin{array}{cc} \sigma_{n,J}^{k} & =\delta C_{n,J}^{k}:\varepsilon_{n,J}^{k}. \end{array}$$

$\delta C_{n,J}$ denotes the reduced secant stiffness tensor of cluster *J*. The algorithmic implementation of the cluster based reduced-order simulation model is analogous to Algorithm Appendix A, except that each cluster of mortar material is now linked with a microscopic BVP.

### 4.2. Numerical Assessment of the Convergence Behaviour of the Reduced Order Simulation (ROS)

In this section, numerical analyses are conducted to investigate the performance of the proposed reduced order multiscale model. The central interests of this study are:

- The convergence behaviour of the ROS in comparison to Direct Numerical Simulations (DNS) on a simple microstructure.
- The convergence behaviour with respect to the number of clusters on a simplified concrete microstructure.

**Figure 6:**
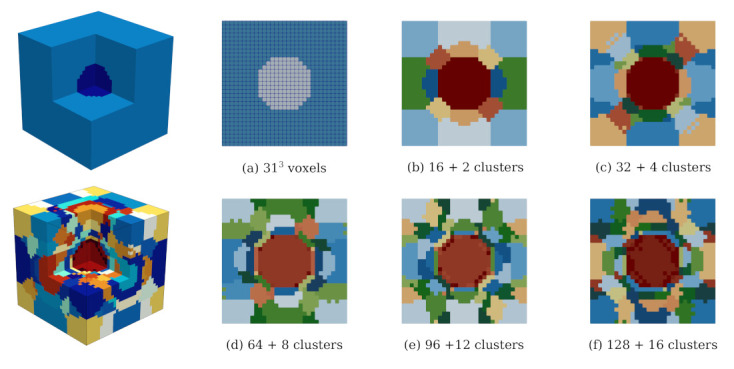
Simple 3D model structure characterised by a cube (size 3.1 cm) with one spherical inclusion at the center and the corresponding clustered microstructures corresponding to various numbers of clusters. The spherical inclusion has the radius of 0.64 cm and occupies a volume fraction of 4.582%. Each color represents a different cluster label.

**Figure 7:**
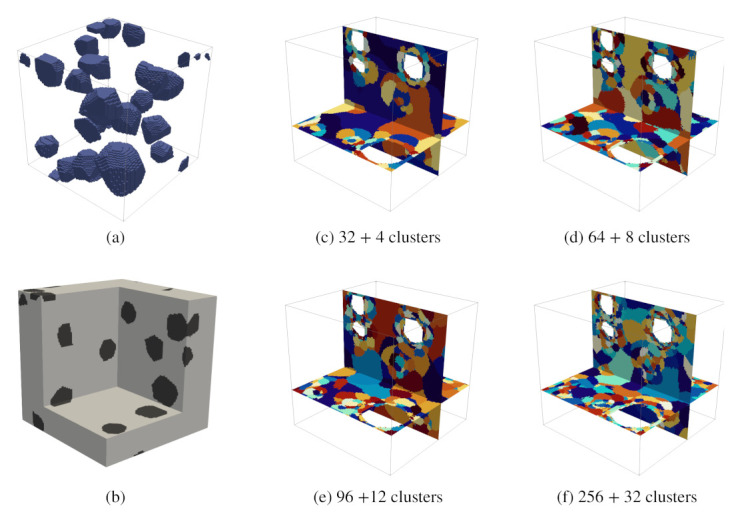
(**a**,**b**) Idealised concrete mesostructures considered in the second analysis. The numerical sample of size 5.05 cm is discretised by $101^{3}$ voxels. The aggregates are modelled using polyhedrons with an aspect ratio ranging from 1 to 1.5 and an average size of 10–15 mm. The inclusion phase occupies a volume fraction of 9.87%; (**c**–**f**) reduced microstructures with 36, 72, 108 and 288 clusters.

Two numerical samples were considered in the analysis. From these microstructures, we generated the corresponding reduced-order microstructures with an increasing number of clusters, as illustrated in Figure 6 and Figure 7. The ratio between cluster count of matrix ($k_{mat}^{ROS}$) and inclusion phases ($k_{agg}^{ROS}$) was chosen to be 8:1. These microstructures are subjected to uniaxial compression with a strain increment of $1\times 10^{-5}$ in the *z*-direction.

To investigate the convergence behaviour of the ROS with respect to the DNS for increasing loading strains, we used the following macrostress-based error criterion

(19) $$\begin{array}{cc} \eta & =\sqrt{\frac{{\left({\tilde{\Sigma }}_{33}-\Sigma_{33}^{ROS}\right)}^{2}}{{\left({\tilde{\Sigma }}_{33}\right)}^{2}}}\times 100\%, \end{array}$$

where ${\tilde{\Sigma }}_{33}$ is the reference macrostress (i.e., the macrostress obtained from the DNS).

#### 4.2.1. Study 1—Comparison with DNS

**Figure 8:**
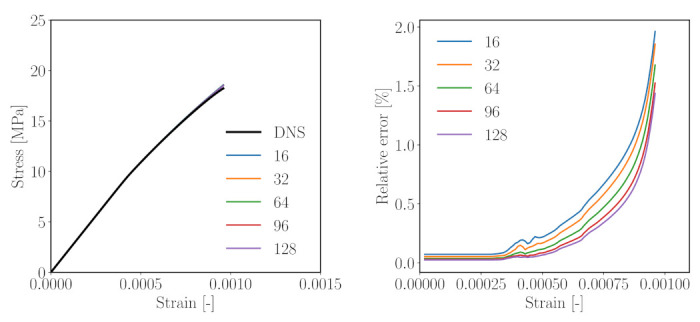
Performance of the k-means-based reduced order scheme in numerical analyses of a simple microstructure subjected to uniaxial compression according to Figure 6. Left: stress–strain curves obtained from five different k-means based models, right: Relative error (measured w.r.t. the direct numerical simulation) of the computed axial stress obtained for a different number of clusters.

Both DNS and reduced-order simulations (ROSs) were carried out for simulations of the simplified mesoscale structure shown in Figure 6. In the case of the ROSs, six unit strain fields were used as training data for the k-means clustering operations. All simulations were run over 100 timesteps. Figure 8 shows the result from the comparative analysis of the simple 3D model structure subjected to uniaxial compression for five numbers of clusters (Figure 6b–f). It can be observed that

- in comparison with DNS, the ROSs capture the overall response in the elastic regime (up to a strain level of 0.03%) well. In this range, an error of only 0.03–0.08% is observed (Figure 8, right).
- The ROSs generally tend to overestimate the computed effective stress as compared to the DNS.
- When microcracking starts in the nonlinear regime, the error increases with increasing loading strain (Figure 8). At the loading strain of 0.1%, up to a 2% error is reported.
- As the number of clusters is increased, the reduced order simulation converges to the result of the high fidelity simulation.

Thus, we can conclude that, for a simple mesostructure, a good convergence can be achieved with the proposed ROS. Even a clustered mesostructure with a small number of clusters can capture the effective behaviour of the composite well. It should be noted that the solution of the uniaxial compression loading on this simplified mesoscale structure using DNS requires computation of a total of 29,791 micromechanical subproblems per load step, as opposed to 16–128 micromechanical evaluations per load step in the case of using ROSs. Even with a theoretical size of $31^{3}$ voxels, DNS requires 164 h running on 300 threads on a high-performance computer, as opposed to approximately 15 h computation time (online stage) on a desktop computer for a reduced model with 144 clusters.

#### 4.2.2. Study 2—Convergence Analysis on a Concrete-Like Meso-Structure

In the second numerical analysis, a concrete-like mesostructural model as shown in Figure 7, subjected to uniaxial compression, is simulated by means of the proposed multiscale reduced order model. The numerical simulation terminates, when the microcrack volume fraction of any given mortar cluster reaches 0.99, which is assumed to be the onset of complete material rupture. Here, the direct numerical analysis was not carried out. Instead, the simulation result of the model with the highest number of clusters is considered as the reference result for the assessment of the error caused by choosing different smaller numbers of clusters.

**Figure 9:**
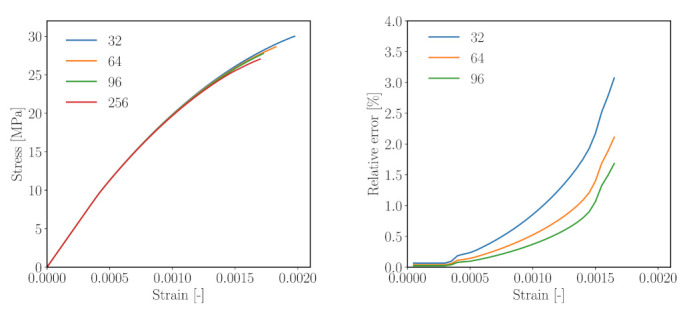
Performance of the k-means-based reduced order scheme in numerical analyses of a simplified concrete meso-structure according to Figure 7 subjected to uniaxial compression. Left: stress–strain curves of four different k-means based reduced samples, right: relative error of the computed axial stress obtained for three different numbers of clusters (Results from 256 mortar clusters taken as reference results).

Five reduced order models with number of mortar clusters $k_{mat}^{ROS}$ = 32, 64, 96 and 256 (corresponding to k= 36, 72, 108, 288, respectively) were considered for the analyses. Herein, the number of clusters of mortar material ($k_{mat}^{ROS}$) is used to denote the numerical result of the associated clustered structure. Figure 9 (left) shows the stress–strain diagrams resulting from the four analyses, and Figure 9 (right) contains the error as a function of the strain level determined for the simulations with $k_{mat}^{ROS}$ = 32, 64, 96 as compared to the result obtained for 256 clusters. It is observed that the error increases with increasing strain level, associated with an increasing microcracking in the microstructure, and that the error decreases with an increasing number of clusters. A larger number of clusters results in an improved resolution of damaging regions in the reduced REV, in particular in the vicinity of aggregates.

Similar to the previous study, one can draw the following conclusions from this study:

- Simulations with a higher number of clusters entered the inelastic stage earlier and failed earlier as well. Subsequently, the maximum compressive stress reduces with increasing cluster count.
- A maximum 3.07% of error with respect to the reference result ($k_{mat}^{ROS}=256$) at ’failure strain’ is recorded.
- In comparison with the previous analysis, the effective stress–strain curves obtained from the different analysis show a slightly larger spread. However, the discrepancy is still within a tolerable range.

In conclusion, with increasing complexity of the microstructure, a higher number of clusters is necessary. However, such choice should also be reasonable, as the computation of the interaction tensor D for each cluster pair is increasingly expensive for finer meshes.

**Figure 10:**
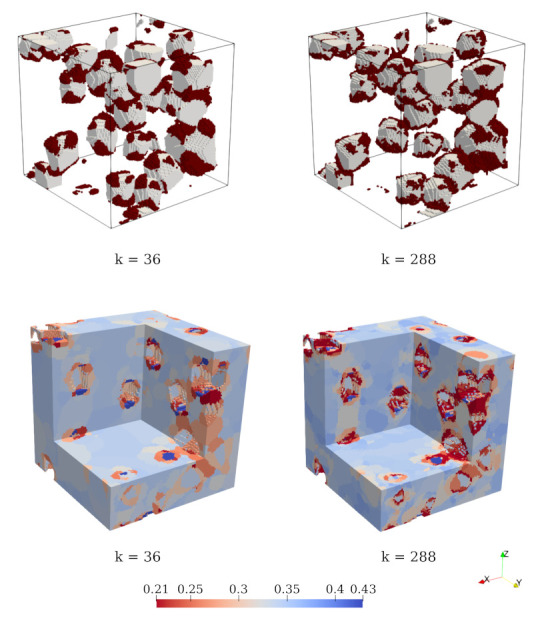
Top left and right: microcracking regions, whose average stiffness is less than 20% of the initial stiffness, obtained from ROS simulations with $k_{mat}^{ROS}$ = 32 and 256. Bottom left and right: visualisation of mortar damage in terms of the distribution of the relative secant stiffness (${\bar{E}}_{rel{,}_{i}}$) at strain = $1.65\times 10^{-3}$ obtained from simulations with $k_{mat}^{ROS}$ = 32 and 256.

It is also of interest to examine further the damage field distribution obtained from the ROS simulations. To characterise damage in the REV, we define a relative stiffness parameter ${\bar{E}}_{rel{,}_{i}}$ as

$$\begin{array}{c} {\bar{E}}_{rel{,}_{i}}=\frac{E_{xx,i}+E_{yy,i}+E_{zz,i}}{3E_{0}}, \end{array}$$

with $E_{rel,i}$ denoting the relative average secant stiffness of the cluster *i* at the current time step. $E_{xx,i},E_{yy,i},E_{zz,i}$ are the current (degraded) Young’s moduli in *x*-, *y*- and *z*-directions, respectively, and $E_{0}$ is the initial Young’s modulus of the mortar cluster *i*. Figure 10 (bottom) visualises the stiffness degradation due to compressive loading computed in two clustered numerical samples. At the same loading strain, the numerical sample with finer discretisation (k=288) yields a higher damage concentration, while it is smeared out in the coarser discretisation. However, one observes that, in both of the fine and coarse discretisations, regions of diffusive damage are qualitatively similar.

#### 4.2.3. Computational Aspects

It is worth pointing out that the computation of the D tensor for each cluster pair in the *offline stage* is computationally intensive. For instance, considering a model of $101^{3}$ voxels, each $D_{IJ}$ tensor took eight seconds of computation time on an Intel(R) Core(TM) i7-8700 CPU @ 3.20GHz with 1.83 GB memory. The code was programmed in Python language. In the online stage, the uniaxial compression simulations of the reduced order model with 34, 108, and 288 clusters over 200 load steps took 2.92, 9.53, and 43 h, respectively. In comparison with the DNS, a substantial speed-up is attained.

## 5. Model Validation: Simulation of a Realistic Concrete Mesoscale Model Subjected to Uniaxial Compression

In this section, a reduced ordered multiscale simulation of a concrete specimen subjected to uniaxial compression is performed. The modelling strategy consists of the following four steps. First, a virtual concrete mesostructure is generated according to the measured aggregate size distribution of concrete (standard AB16). Second, the material properties of the material constituents are specified. The third step is the determination of the localisation tensors for each voxel in the mesostructure for the k-means based domain decomposition procedure (*offline stage*). Finally, a simulation of the mesoscale model subjected to uniaxial compression is performed on a synthetic concrete sample, and the model predictions are compared with experimental data (*online stage*).

### 5.1. Simulated Concrete Sample

**Figure 11:**
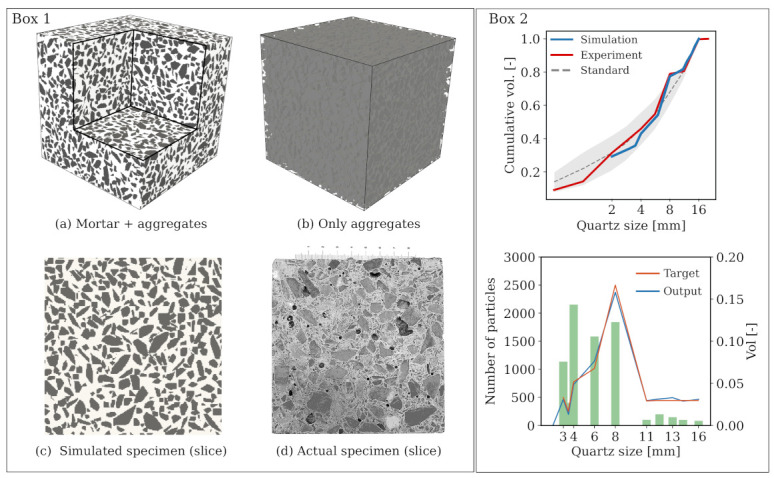
Box 1: Visualisation of the numerical concrete sample representing a specimen of size 10 cm (top), and qualitative comparison between slices of virtual and actual samples (bottom), Box 2: Cumulative volume distribution of aggregates (top) and size distribution of the quartzitic particles (bottom).

A virtual concrete mesostructure corresponding to the concrete standard AB16 is generated using PyCMG [65], an opensource concrete mesostructure generator. The sample consists of $201^{3}$ voxels with the smallest and largest aggregate sizes of 3, and 16 mm, respectively. In total, an approximate 47.75% coarse aggregate is explicitly resolved and thus the remaining 22.25% fine aggregate content (<3 mm) is implicitly incorporated into the mortar matrix via continuum micromechanics homogenisation. Figure 11 (bottom) shows a comparison of the synthetically generated concrete mesostructure and the actual concrete specimen. It is noteworthy that it takes only 201 s on a standard computer to generate the above virtual specimen. For interested readers, further details regarding the generation procedure involving geometrical parameters as well as the realistic aspects of the virtual concrete specimen are described in [68].

### 5.2. Calibration of the Parameters of Mortar Constituents

**Figure 12:**
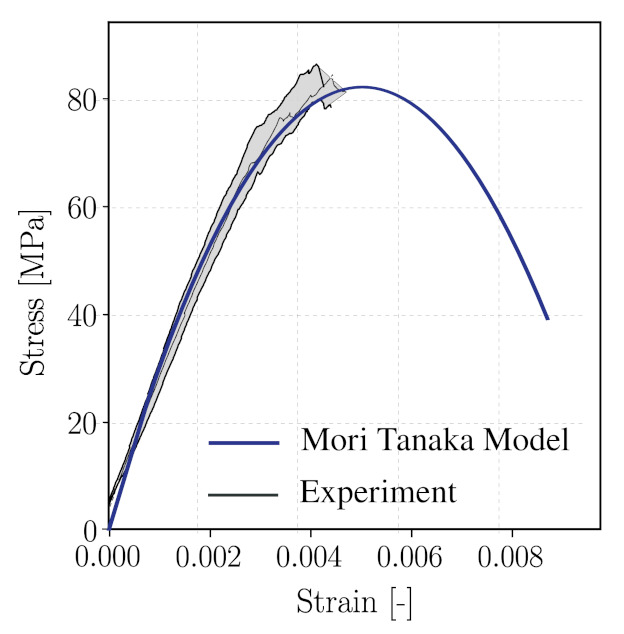
Comparison of the calibrated stress–strain curve with the experimental data of a mortar sample ($\varphi_{quartz}=30.34\%$). The micromechanics based model predicts the initiation of microcracking in mortar sample at a strain level of $8\times 10^{-4}$.

At the mesoscale, while the material properties of the aggregates are measured directly in laboratory tests, the material properties of the considered mortar matrix is to be determined. To this end, using the material data obtained from the experiment, we proceed to calibrate the model parameters at the constituents of mortar, i.e., the cement paste and the fine aggregates, according to the following calibration strategy:

- We use experimental data from the mortar samples ($\varphi_{quartz}=30.34\%$) to calibrate the microscopic constituents parameters such that the Young’s modulus and compressive strength of the mortar REV match the experimental stress–strain curve. The parameters to be calibrated include the Young’s modulus of the cement paste solid $E_{s}$, the microcrack volume fraction $\varphi_{c}$ and their aspect ratio *X*, and the microscopic fracture energy $g_{f}$. Figure 12 shows the calibrated (homogenised) stress–strain curve of the mortar REV in comparison with the experimental stress–strain curve.
- In the numerical concrete sample (Figure 11a), the mortar matrix contains cement paste and fine aggregates with the relative proportion of $\frac{\varphi_{cem}}{\varphi_{quartz}}=\frac{29.56}{22.25}$ between the two material phases. Thus, $\varphi_{quartz}$ in mortar matrix equals 43.95% and differs from the actual mortar sample. We assume the content of microcracks is identical for the same cement mixture. The effective Young’s modulus of mortar matrix is obtained, by setting $\varphi_{quartz}=43.95\%$ instead of 30.34%, while the other parameters are kept unchanged. As a result, the mortar matrix of the simulated concrete has a Young’s modulus of 30.05 GPa. At this scale, the fine sand grain inclusions are assumed to have a spherical geometry. A summary of the parameters is contained in Table 5.

In Table 5, the parameters listed in the group *Material parameters* are obtained from the laboratory investigation reported in Section 2. As the name indicates, *Model parameters* lists micromechanics parameters, which are to be calibrated. It is worth mentioning that the Young’s modulus and the Poisson ratio of cements paste solid is referred to as the elastic parameters of the theoretical intact cement solid without microcracks. Thus, these values are higher than the experimental range for real hardened cement paste with pre-existing microcracks. By introducing microcracks ($\varphi_{c}=8.3\%$) and fine aggregates (φ=30.34%), the effective properties of mortar at zero-stress state reduces to 29.8 GPa and 0.124, as shown in the group *Homogenised parameters*. These values match the experimentally measured range of values for mortar (Figure 12).

It is also of interest to estimate the effective elastic properties of the numerical concrete sample, given that the morphology of the mesostructure and the elastic properties of all constituents are known. A FFT-based computational homogenisation was performed on the numerical concrete sample. From the resulting effective stiffness tensor, we obtained effective material constants at the concrete level listed in Table 6. In comparison with the laboratory test, the Young’s modulus of the virtual concrete sample is approximately 4.7% larger, which is considered as satisfactory agreement. The Poisson’s ratio is slightly smaller as compared to the test. Due to the stochastic arrangement of the aggregates in the synthetic concrete sample, a slight anisotropy of the Young’s modulus in axial and lateral direction with a ratio of 1.003 is obtained.

### 5.3. Offline Training Stage

**Figure 13:**
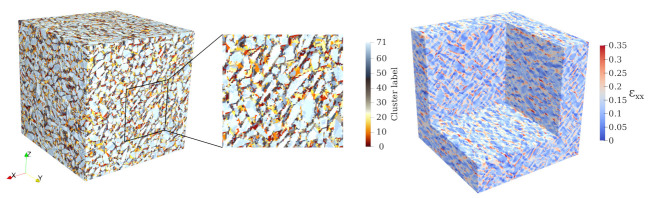
Left and center: Visualisation of the clustered mesostructure of the virtual concrete specimen obtained from the k-means cluster algorithm, Right: One of the strain components ($\varepsilon_{xx}$) that was used to compute the clusters shown in the image on the left, the red color indicates high values of positive strain components, which correlates with microcracking.

In the *offline stage*, we used the six unit strain fields obtained from the FFT-based homogenisation step as training data to evaluate the similarity in the mechanical behaviour of all voxels. Intuitively, two additional strain fields as training data were included in the k-means clustering step, as suggested in [55]: (i) the local strain field under uniaxial compression in the *z*-direction (see Figure 13 right), and (ii) the positive component of the strain field during uniaxial compression in the *z*-direction. In this numerical analysis, the high fidelity mesostructure is decomposed into 72 clusters, with the ratio between the number of matrix clusters and the inclusion clusters chosen as 8:1. This is motivated by the priority of capturing the damage evolution in the mortar matrix (Figure 13 (left, center)).

This choice of the cluster configuration results in the computation of $72^{2}=5184$ cluster pairs. An average computation time for each $D_{IJ}$ tensor took 55 s and 17.3 Gigabyte RAM. In our implementation, the computation of interaction tensors was performed in parallel on a high performance cluster using 50 Intel(R) Xeon(R) Gold 6148 CPU @ 2.40 GHz. The total computational time was approximately 43 min.

### 5.4. Simulation of a Uniaxial Compression Test on the Virtual Concrete Sample

With the preparations accomplished in the offline stage, simulation on the reduced mesostructure subjected to uniaxial compression can be performed. The uniaxial load condition is simulated by restricting the macroscopic strain component $E_{zz}$ to a prescribed value, and setting the macroscopic stress components perpendicular to the *z*-direction to zero. The strain increment is prescribed as $1\times 10^{-5}$. The simulation terminates when the microcrack volume fraction of one cluster reaches 0.99, which is assumed to be the onset of complete failure in material. It is to be noted that the multiscale homogenisation method, by following the scale separation principle [71], is restricted to the pre-peak regime of concrete. Possible remedies are also proposed in [72,73,74]. However, it is beyond the scope of the study.

The macroscopic stress tensor at load level E is recorded and evaluated as

$$\begin{array}{c} \Sigma_{\mathrm{Macro}}=\sum_{1}^{Nclusters}c_{I}\sigma_{I}, \end{array}$$

where $c_{I}$ and $\sigma_{I}$ are the volume fraction and mesoscopic stress tensor of a cluster *I*, respectively.

**Figure 14:**
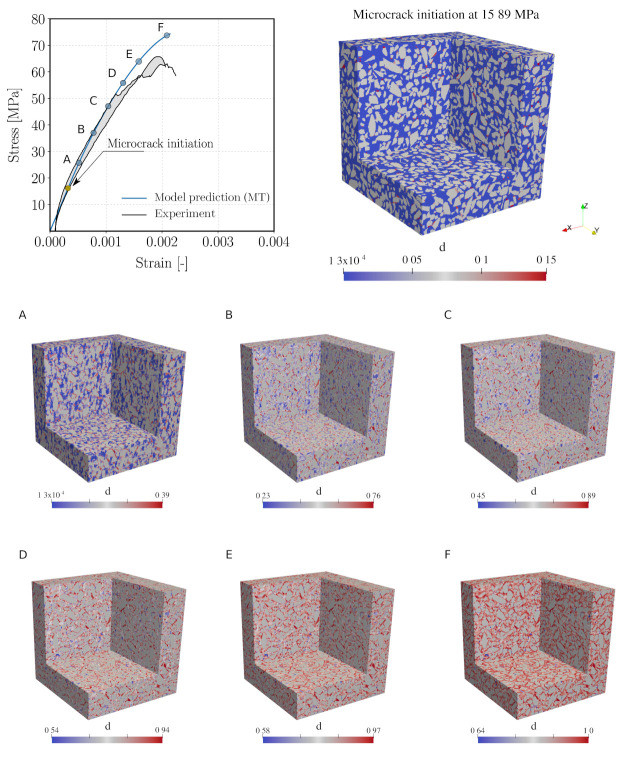
Top left: Stress–strain curves obtained from the simulation (blue line) and the experiment (black lines), Top right: Visualisation of damage distribution at a microcrack initiation state. From (**A**–**F**): Opening of vertically oriented microcracks in the mortar matrix at six different load levels (25.77, 37.05, 47.06, 55.88, 63.41, 73.71 MPa, red color indicates active microcracking regions).

Figure 14 (top left) shows the stress–strain curve predicted from the reduced order multiscale model and a comparison with experimental data. We can observe that the initial portion of the curve up to approximately 50% of the measured compressive strength agrees well with the measure curve. However, the proposed multiscale model leads to an overestimation by 13.4% of the material strength. Several reasons may be responsible for this deviation. One reason possibly lies in the calibration of the parameters for the mortar matrix according to measurements of separate mortar specimen, which are characterised by a different volume fraction of fine aggregates as compared to the one used in the concrete specimen (see also respective comments in Section 5.2). Another possible cause may be connected with the influence of initial defects at mesoscale, in particular the interfacial transition zone (ITZ), which play a significant role in the damage process in concrete. The ITZ, however, is not yet separately considered in the current modelling approach. Finally, the choice of the analytical homogenisation scheme at the microscale (currently, the Mori–Tanaka scheme) might play a role. The role of the micromechanics homogenisation scheme will be further investigated in Section 5.6.

### 5.5. Interpretation of Numerical Results

To examine the evolution of the stiffness degradation due to microcracking, the effective secant stiffnesses in three orthogonal directions were recorded for all clusters at each load step. To show the effect of microcracking on the transversal stiffness degradation of each cluster, we introduce the damage parameter $d_{lat}$:

$$\begin{array}{c} d_{lat}=1-\frac{0.5(E_{xx}+E_{yy})}{E_{0}}, \end{array}$$

where $E_{xx}$ and $E_{yy}$ are the current secant Young’s moduli in *x* and *y*-directions, respectively, and $E_{0}$ denotes the initial concrete stiffness. $d_{lat}=0$ indicates no damage, and $d_{lat}=1$ denotes a complete loss of transversal stiffness of the respective cluster.

From the stress–strain curve (Figure 14 (top left)), it can be seen that the initial microcracks start propagating at a strain level of $3.153\times 10^{-4}$, corresponding to a loading level of 15.89 MPa (20.37% of the compressive strength). In contrast, damage initiation in the calibrated mortar REV is recorded at a much later stage (ε = $8\times 10^{-4}$). This indicates that, due to the heterogeneity of the material, the threshold value of strain state that triggers propagation of existing microcracking is reached already at a relative low loading level of loading in several regions of the mesostructure. Such early initiation of damage is also confirmed by several non-destructive tests on concrete [75,76]. Figure 14 (top right) highlights the regions exhibiting the start of microcrack propagation.

**Figure 15:**
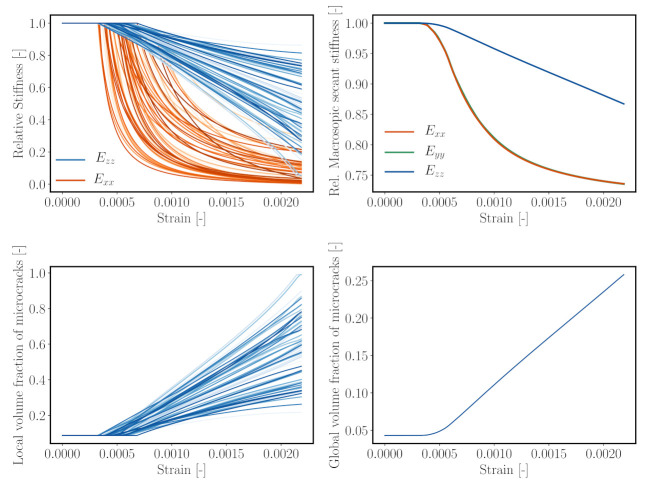
Top left: relative stiffness evolution under uniaxial compression of all mortar clusters in *x*- and *z*-directions. Top right: relative macroscopic secant stiffness of concrete under uniaxial compression at macroscopic scale. Bottom: volume fraction of microcracks, in all mortar clusters (left), and total volume fraction of microcrack in concrete samples (right).

Figure 14A–F illustrates the damage evolution in *x*- and *y*-directions with increasing loading level. All clusters connected with the mortar material exhibit a gradually increasing growth of microcracks parallel to the loading direction, leading to an anisotropic reduction in the macroscopic secant stiffness. This is corroborated by Figure 15 (top), which shows the evolution of the secant stiffnesses both in axial (*z*) and lateral (*x*) directions. As seen in Figure 15 (top left), the lateral stiffnesses (in *x*- and *y*-directions) of all clusters decrease at a much faster rate in comparison with the longitudinal one due to the growth of microcracks, whose orientation is parallel to the major stress axis. Moreover, a closer inspection into the spatial distribution of damage in the mortar matrix reveals that microcracking takes place predominantly in the immediate vicinity of the aggregates, caused by the stress concentration in these regions of the mesoscale structure. In addition, these degradation mechanisms revealed by the multiscale reduced order model is in agreement with experiment observations [75].

Recording the microcrack volume fraction provides information on the damage evolution in the clusters as well as in the complete mesostructure of the virtual concrete specimen. Figure 15 (bottom left) shows the evolution of the microcrack volume fraction in all clusters during compressive loading. At ultimate load, the microcrack volume fraction in one cluster reaches a value of 1. Comparing Figure 15 (bottom left) and (bottom right), one observes that this state corresponds to a 25% total microcrack volume fraction in the virtual concrete sample.

**Figure 16:**
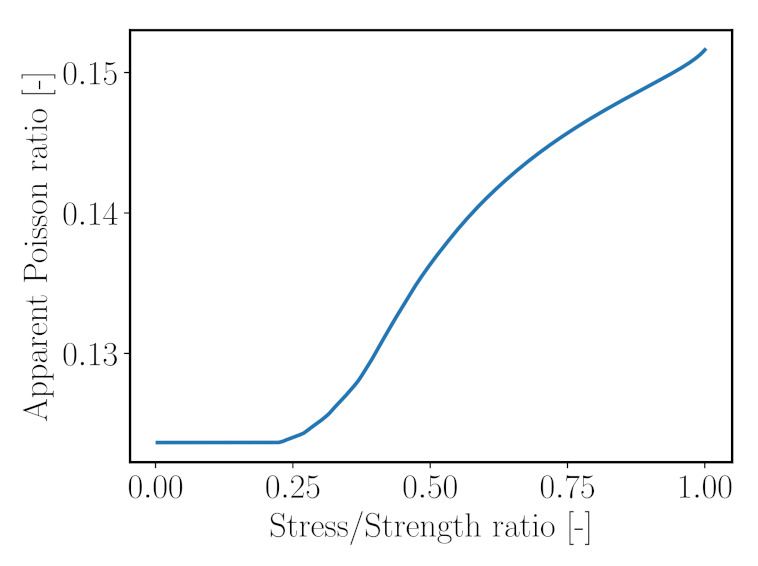
Predicted evolution of the apparent Poisson ratio with increasing load level.

Once microcracking initiates, the model is also able to predict an increase of the apparent Poisson’s ratio, defined as the ratio between the transversal and axial strain,

(20) $$\begin{array}{c} \nu_{macro}=-\frac{\varepsilon_{macro,xx}}{\varepsilon_{macro,zz}}, \end{array}$$

(Figure 16). It shows a nonlinear increase of the Poisson’s ratio as soon as microcracks are starting to propagate. This effect also was observed in laboratory tests [77].

### 5.6. Improvement of the Model by Means of the Modified Interaction Direct Derivative Scheme (MIDD)

As already discussed above, the choice of the homogenisation scheme for the mortar matrix material at the microscale may have a strong influence on the predicted response of concrete at the macroscopic scale. As can be seen in Figure 5 and Figure 12, in the post-peak regime, the reduction of stiffness in the longitudinal direction is relatively steady, which results in a prolonged stress–strain curve. An overestimation of concrete compressive strength can be attributed to the Mori–Tanaka homogenisation scheme as the matrix material is always assumed to be “connected” and the spatial distribution mimics that of the inclusion shape [78].

To replicate a more brittle behaviour of the mortar matrix, we now investigate using the Interaction Direct Derivative homogenisation scheme [79] (IDD) in this subsection as an alternative scheme. The IDD scheme allows consideration of not only the microcrack morphology, but also the spatial distribution of microcracks, which is proven to play a significant role in damage behaviour of cracked solids (see, e.g., [29]).

**Figure 17:**
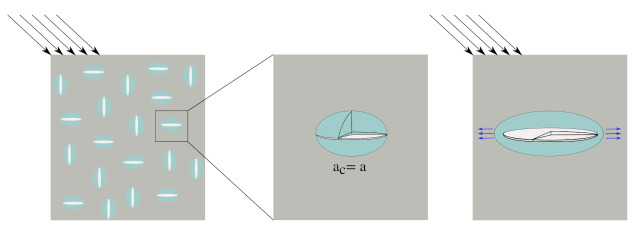
Schematic illustration of the IDD homogenisation scheme.

The distribution of the microcracks is determined by the shape of a “double cell” surrounding a microcrack (see Figure 17). The shape of the double cell surrounding a crack has a clear physical meaning and often is idealised as an ellipsoid. In our model, we assume that the microcrack distribution takes the form of an oblated spheriod and is coaxial with the associated microcracks, as illustrated in Figure 17. Given three microcrack families with radius *a*, half thickness *c* and crack density N, the geometrical parameters characterising the double cell for a certain microcrack family *i* are the double cell radius $a_{D}$ and the half thickness $c_{D}$. We denote $X_{D,i}=\frac{a_{D}}{c_{D}}$ as the aspect ratio of the cell. An explicit formulation [80] for estimating the stiffness is given as

(21) $$\begin{array}{cc} C_{hom}^{IDD} & =C_{mat}+{\left(I^{s}-\sum_{i}^{3}\varphi_{i}A_{i}^{dil}:P_{i}^{D}:\left(C_{i}-C_{mat}\right)\right)}^{-1}:\sum_{k}^{3}\varphi_{k}\left(C_{k}-C_{mat}\right):A_{k}^{dil}, \end{array}$$

(22) $$\begin{array}{cc} A_{i}^{dil} & ={\left(I^{s}+P_{i}:\left(C_{i}-C_{mat}\right)\right)}^{-1}, \end{array}$$

(23) $$\begin{array}{cc} P_{i}^{D} & =S_{i}^{D}:D_{m}, \end{array}$$

where $P_{i}^{D}$ is the Hill Polarization tensor corresponding to the double cell *i*. The superscript *D* denotes quantities belonging to the distribution (i.e., the double cell) of the microcracks. When the geometry of the distribution is identical to that of the microcracks, the prediction of the IDD and Mori–Tanaka schemes is identical. Assuming zero stiffness for the three crack families yields

(24) $$\begin{array}{cc} C_{hom}^{IDD} & =C_{m}-{\left(I^{s}+\sum_{i}^{3}\varphi_{i}{\left(I^{s}-S_{i}\right)}^{-1}:S_{i}^{D})\right)}^{-1}:\sum_{k}^{3}\varphi_{k}C_{m}:{\left(I^{s}-S_{k}\right)}^{-1}. \end{array}$$

When the external load reaches a critical value, pre-existing microcracks start propagating. As the microcrack distribution in the IDD model is governed by the double cell that encloses the microcrack, we assume that this double cell evolves to accommodate the microcrack. Eventually, the microcrack distribution flattens and follows the penny shape of microcracks. The concept of an evolving microcrack distribution was suggested by various analyses, such as [29,79,80]. It is found that, in order to capture the characteristic softening behaviour of mortar, (i) the growth of the double cell has to be in proportion with the ratio between $X_{i}$ and $X_{D,i}$, and (ii) the growth rate of the double cell is higher than the growth rate of crack family *i*.

To control the growth of double cell $D_{i}$, we introduce a dimensionless parameter κ. When subjected to an applied macrostrain E, the current crack radius $a_{i}$ is evaluated according to the procedure described in Section 3.2.1. The growth of the double cell determining the microcrack distribution is computed as

(25) $$\begin{array}{c} a_{D,i}=a_{i}\frac{X_{i}}{X_{D,i}}\kappa_{i},\;\;\left(\kappa_{i}>1\right). \end{array}$$

A calibration procedure was undertaken to obtain parameters for the double cell i.e., the microcrack distribution, which are able to realistically replicate the response of a mortar sample subjected to uniaxial compression. According to this calibration procedure, the initial aspect ratio $X_{D}$ and distribution growth rate κ of crack distribution are found to be 12 and 1.05, respectively. All remaining microscale parameters are kept unchanged and listed in Table 5.

**Figure 18:**
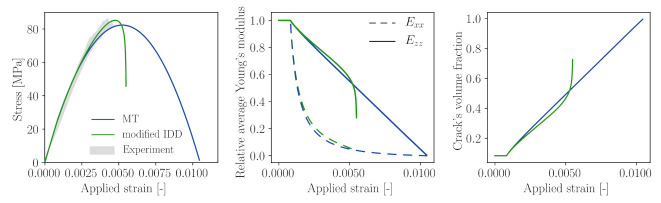
Left: Computed stress–strain response of mortar subjected to uniaxial compression using the calibrated MIDD scheme (green) in comparison with the experimental range (grey) and calibrated Mori–Tanaka scheme (blue). Center: Evolution of stiffnesses in *x* and *z*-directions, respectively. Right: Evolution of microcrack volume fraction.

Figure 18 shows the stress–strain diagram of mortar sample obtained from the model using the modified IDD estimated with the calibrated double-cell parameters. In comparison with the Mori–Tanaka estimate (MT) (also shown in Figure 18), we see that a more brittle behaviour of mortar is obtained from the MIDD scheme. As this experiment was used for calibration, the compressive strength is well replicated. In the post-peak regime, a brittle response is predicted. It should be noted that the simulation terminates as the point where the homogenised stiffness becomes negative, predicted by the MIDD scheme. As soon as the stress–strain curve enters the softening branch, a sudden drop in stress is observed. Using the modified IDD scheme predicts failure of the mortar material at a microcrack volume fracture of 0.7, while, in the MT estimate, the material fails at a microcrack volume fraction 1.

The modified IDD scheme has also been applied to simulations of the virtual concrete sample subjected to uniaxial compression, which has been investigated before in Section 5 using the MT homogenisation scheme on the microscale of the mortar matrix. The parameters for the double cell calibrated before for mortar material are used, and all remaining parameters are identical to those listed in Table 5.

**Figure 19:**
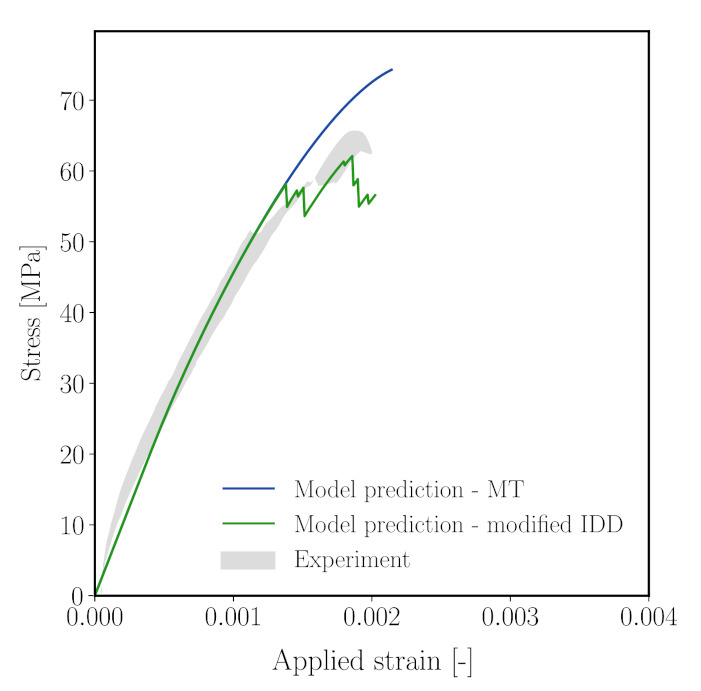
Stress–strain response of concrete numerical sample obtained from Mori–Tanaka scheme (blue), modified IDD scheme (green), in comparison with the experimental range (grey).

The stress–strain diagram obtained from this modified micromechanics-reduced order meso-scale model for concrete is shown in Figure 19 and compared with the previous result from the model using the Mori–Tanaka scheme and the experimental results. Now, the peak load is in much better agreement with the compressive strength of the concrete sample recorded in the laboratory, with a deviation of only 3.52%. Interestingly, above 93% of the peak load, the stress–strain curve is no longer continuous. This is attributed to a combination of disorder at the mesoscale and the rapid reduction in longitudinal stiffness after peak stress (Figure 18 center).

## 6. Conclusions

In this work, a reduced order multiscale model for computational simulations of distributed microcracking in concrete on the mesoscale has been presented. Within the mortar matrix, a continuum micromechanics model takes into account pre-existing microcracks, which may propagate according to the Griffith criterion. This continuum micromechanics model in conjunction with a fracture energy model for crack propagation was incorporated into a computational mesoscale model of concrete. At this scale, a numerical model is proposed, which realistically resolves the coarse aggregates and its size distribution. To reduce the computational costs for high resolution multiscale simulations, a k-means based model reduction technique [53] has been employed. First, a synthetic concrete mesostructure has been generated from the size distribution of coarse aggregates determined in the laboratory. Next, the parameters of the micromechanics based microcracking model that governs the behaviour of the mortar material have been calibrated using experimental data for the individual constituents, i.e., the cement paste, the fine aggregates in the mortar matrix, the initial microcrack volume fraction in the initial state (due to autogeneous shrinkage), and the coarse aggregates. Finally, the two scale reduced order concrete model has been validated by means of data from uniaxial compression tests performed in the laboratory. Based on the results, the following conclusions can be drawn:

1. It was observed that the Mori–Tanaka scheme, which has been used in the initial model design at the micro-level, overestimates the compressive strength of concrete.
2. In order to improve the model predictions, the Mori–Tanaka scheme governing microcracking at the microscale has been replaced by an improved interaction direct derivative scheme [79]. This model is able to incorporate information on the microcrack distribution, which, however, needs additional calibration effort.
3. After calibration of the additional distribution-related parameters, model predictions of the improved model for the uniaxial compression test have substantially improved, with a deviation of only 3.2%.
4. The proposed model has been proven to be capable of simulating anisotropic microcrack evolution, leading to anisotropic stiffness degradation on the macroscopic level. In addition, the evolution of the Poisson’s ratio during loading could be predicted.

Nevertheless, the model should also be extended to account for the interfacial transition zone (ITZ). This will enable the investigation of the effect of ITZ on the overall behavior of concrete. Lastly, two potential practical applications are also outlined:

1. Due to the multiscale nature of the proposed model, it can be used to simulate a wide variety of concrete compositions by simply altering the predominantly physically measurable microscale and mesoscale parameters governing the topology and material properties of the required concrete composition. Thus, the proposed modelling framework can be the basis of a Virtual Material Testing Environment and can collaboratively aid in the development of concrete with a better performance.
2. From a structural health monitoring view point, the outcome of the model can serve as a high-fidelity input for an ultrasonic-wave propagation numerical investigation of damaged concrete (see, e.g., [81,82,83]). This, in turn, can support the development of an ultrasonic wave based technology (the so-called coda wave) on early detection of (diffuse) damage in concrete.

## Figures and Tables

**Table 1:** Composition of concrete standard AB16 used in the experiments.

Type	Description	Amount	Density	Volume
[-]	[-]		[kg/dm${}^{3}$]	[dm${}^{3}$/m${}^{3}$]
w/c		0.45		
Cement	CEM I 52.5 R	350 [kg/m${}^{3}$]	310	112.9
Plasticizer		1.0 [m-%]		
Air voids		2.00 %	0	20
Water		157.5 [kg/m${}^{3}$]	1	157.5
Aggregate 0/2	Quartz	39.46 [%]	2.67	280.02
Aggregate 2/5	Quartz	12.18 [%]	2.64	86.44
Aggregate 5/8	Quartz	28.91 [%]	2.64	205.13
Aggregate 8/16	Quartz	19.45 [%]	2.65	138.01

**Table 2:** Experimental quantification of the concrete compositions according to standard AB16.

	Cement Matrix	Fine Aggregates						Coarse Aggregates					
**Size [mm]**	**-**	**0.063**	**0.125**	**0.25**	**0.5**	**1**	**2**	**2.8**	**4**	**5.6**	**8**	**11.2**	**16**
**Volume fraction [%]**	29.259	1.504	1.619	1.758	1.758	3.634	12.174	5.0626	5.146	6.743	16.606	2.904	11.832
**Total [%]**	29.259	22.448						48.292					
**Total [%]**	**29.259**	**70.741**											

**Table 3:** Young’s Modulus, Poisson’s ratio, and the compressive strength of the quartzitic aggregates, mortar, and the concrete with an AB16 grading curve.

Material Parameter	Quartzitic Aggregate	Mortar	AB16 Concrete
**Young’s Modulus E [GPa ]**	84.6	27.1	48.03
**Poisson’s ratio ν [-]**	0.12	0.19	0.15
**Compressive strength $f_{c}$ [MPa]**	368	80.3	64.4

**Table 4:** Summary of parameters used for the analysis of a mortar REV subjected to uniaxial loading. The following sources have been used to determinate the parameters: ${}^{1}$ —Experiment (Section 2), ${}^{2}$—[25], ${}^{3}$—[64].

**Material parameters**			
Young’s modulus of aggregates ${}^{1}$	$E_{agg}$	84.6	GPa
Poisson ratio of aggregates ${}^{2}$	$\nu_{agg}$	0.2	
Aggregates’ volume fraction	$\varphi_{agg}$	0.35	
**Model parameters**			
Microcrack’s initial radius ${}^{2}$ (*i* = 1, 2, 3)	$a_{0,i}$	0.017	mm
Microcrack’s initial half thickness ${}^{2}$	$c_{0,i}$	0.001	mm
Microcrack’s density ${}^{2}$	N	$3.241\times 10^{4}$	1/mm
Young’s modulus of cement paste solid	$E_{s}$	44	GPa
Poisson ratio of cement paste solid ${}^{\begin{array}{c} named-contentcolortypergbcontent-type0,0,03 \end{array}}$	$\nu_{s}$	0.25	
Mortar solid microscopic fracture energy	$g_{f}$	6.88	N/mm
**Numerical output**			
Young’s modulus of mortar REV	$E_{mortar}$	26.9	GPa
Compressive strength of mortar REV	$f_{c}$	54.68	GPa
Tensile strength of mortar REV	$f_{t}$	3.68	MPa
Ratio between Compressive and Tensile strength	$\frac{f_{c}}{f_{t}}$	14.86	

**Table 5:** Material parameters for the large quarzitic aggregates and the calibrated parameters for mortar, containing a volume fraction $\varphi_{quartz}=30.34\%$ of fine quarzitic aggregates. The mortar matrix is assumed to contain a 8.3% volume fraction of initial microcracks with an initial aspect ratio of 23. In the investigated concrete sample, the material properties of the mortar matrix with a volume fraction of $\varphi_{quartz}=43.95\%$ are determined by varying the volume fraction of aggregate accordingly in the Mori–Tanaka based homogenisation procedure, while keeping other parameters unchanged (group *Model parameters*). The following sources are used to determine the parameters: [${}^{1-3}$]—Experiment, [${}^{4,5}$] aspect ratio is taken within the range measured in [69], ${}^{6}$—[24,70].

**Material parameters (from laboratory tests)**			
Young’s modulus of aggregates	$E_{agg}$	86.4	GPa
Poisson ratio of aggregates	ν	0.12	
Volume fraction of aggregates	$\varphi_{agg}$	0.3034	
**Model parameters**			
Microcrack initial radius (*i* = 1, 2, 3)	$a_{0,i}$	0.023	mm
Microcrack initial half thickness	$c_{0,i}$	0.001	mm
Microcrack’s density	N	$1.25\times 10^{4}$	1/mm${}^{3}$
Mortar solid microscopic fracture energy	$g_{f}$	6.61	N/mm
Young’s modulus of cement paste solid	$E_{s}$	49	GPa
Poisson ratio of cement paste solid	$\nu_{s}$	0.23	
**Homogenised parameters**			
Young’s modulus of mortar	$E_{mortar}$	29.8	GPa
Poisson ratio of mortar	ν	0.124	
Compressive strength of mortar	$f_{c}$	81.34	MPa

**Table 6:** Homogenised elastic properties of the synthetic concrete sample in comparison with experimental data obtained from a uniaxial compression test.

Volume Fraction [%]	Average Modulus of Elasticity [GPa]		Poisson’s Ratio [-]		Anisotropy [-]
	Model	Exp.	Model	Exp.	
47.74	50.311	48.03	0.124	0.152	1.003

**Figure A1:**
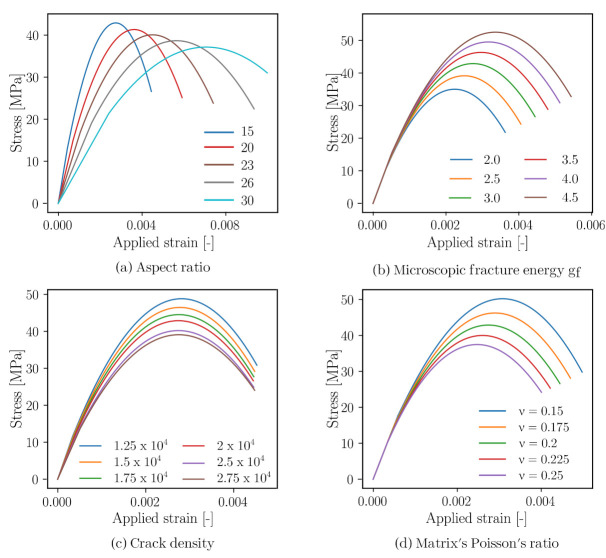
Parametric study: effect of model parameters at a microscopic scale on the compressive response of mortar REV.

## Data Availability

Data sharing is not applicable.

## Abbreviations

The following abbreviations are used in this manuscript:

CWI	Coda Wave Interferometry
FFT	Fast Fourier Transform
REV	Representative Elementary Volume
LEFM	Linear Elastic Fracture Mechanics
CMG	Concrete Mesostructure Generator
DNS	Direct Numerical Simulation
ROS	Reduced Order Simulation
SCA	Self-consistent Clustering Analysis
ITZ	Interfacial Transition zone
IDD	Interaction Direct Derivative
MIDD	Modified Interaction Direct Derivative

## Appendix A. Structure of Hierarchical Numerical Simulation of the Multiscale Model

**Algorithm 1:** Structure of hierarchical numerical simulation of the multiscale model (High-fidelity model).

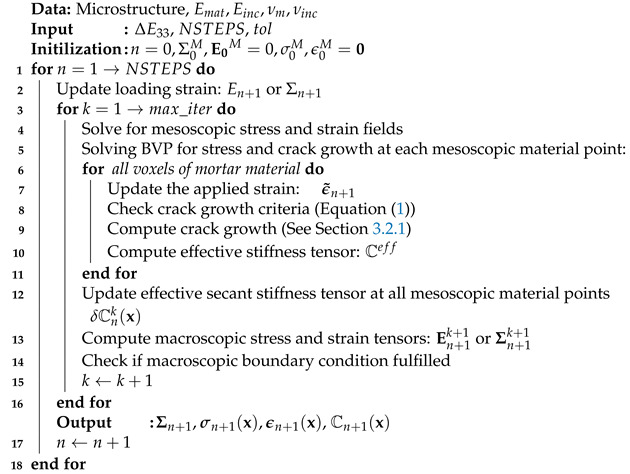

## Appendix B. Parametric Study of Mori–Tanaka Based Damage Model of Cementitious Composite

In this analysis, we investigate the effect of the initial aspect ratio *X*, the microscopic fracture energy $g_{f}$, the crack density N, and the Poisson ratio of the mortar solid $\nu_{s}$. Here, the mortar solid refers to the mortar composite without the microcracks. Given the microcrack volume fraction and the microcrack geometry, the effective stiffness of the mortar REV can be computed using the Mori–Tanaka homogenisation scheme and is given by the following expression:

(A1) $$\begin{array}{c} C^{eff}=C_{m}-\sum_{i=1}^{3}\varphi_{cr,i}C_{m}:A_{MT,i}, \end{array}$$

and the effective stress as the mesoscale is evaluated using

(A2) $$\begin{array}{cc} \sigma & =C^{eff}:E. \end{array}$$

Here, $A_{MT,i}$ is the Mori–Tanaka strain localisation tensor of microcrack family *i*,

(A3) $$\begin{array}{cc} A_{MT,i} & =A_{D,i}:{\left(A_{D,i}\varphi_{cr,i}+I^{s}\varphi_{m}\right)}^{-1}, \end{array}$$

(A4) $$\begin{array}{cc} A_{D,i} & ={\left(C_{m}-C_{cr,i}\right)}^{-1}:C_{m}:{\left({\left(C_{m}-C_{cr,i}\right)}^{-1}:C_{m}-S_{cr,i}\right)}^{-1}. \end{array}$$

Assuming zero stiffness of all microcrack families, Equation (A4) is simplified as

(A5) $$\begin{array}{cc} A_{D,i} & ={\left(I^{s}-S_{cr,i}\right)}^{-1}. \end{array}$$

The parameter range chosen for the subsequent study is listed in Table A1.

**Table A1:** Summary of parameters used for the parametric study.

Model Parameters		
$a_{0,i}$	15,20,23,26,30	μm
$c_{0,i}$	1	μm
N	1.25,1.5,1.75,2,2.6,2.75	$\times 10^{4}/{\mathrm{mm}}^{3}$
$g_{f}$	2,2.5,3,3.5,4,4.5	N/mm
$E_{solid}$	43	GPa
$\nu_{solid}$	0.3	

The first analysis is regarding the influence of the aspect ratio. In the micromechanical model, the aspect ratio is controlled by modifying the initial crack radius, while crack thickness is held constant in all simulations. In general, the absolute size of a penny-shaped microcrack is irrelevant, at least from the point of view of the mathematical formulation. The choice of the microcrack dimensions should nevertheless follow the principle of the *separation of scales*. The effect of aspect ratio is taken into account via the computation of the internal Eshelby tensor for that associated crack family. As can be seen in Figure A1 (top-left), by increasing the size of the microcrack radius, a reduction in the compressive strength is observed.

Moreover, due to the increase in the microcrack radius, the initial volume fraction of microcracks is also higher, thus it leads to a significant reduction in the initial stiffness of the mortar REV. We see that mortar whose microcrack’s aspect ratio of higher value exhibits a more prolonged nonlinear stress strain curve. This effect is not trivial and can be explained as follows: an increase in the aspect ratio leads to a reduction of the Poisson ratio of the microcracked mortar, according to the Mori–Tanaka homogenisation scheme. Thus, in uniaxial compression, given a constant applied strain, the ratio between the positive components of strain tensor and the maximum strain components is smaller, thus the crack grows at a much slower rate.

The influence of the microscopic fracture energy parameter $g_{f}$ is shown in Figure A1 (top right). A smaller value of $g_{f}$ yields an earlier microcrack initiation and, subsequently, lower compressive strength. The elastic property or the REV is not affected by this parameter.

Meanwhile, a high value of microcrack density N has a negative effect on both initial stiffness and the compressive strength. However, the parameter is insensitive to the strain value at peak stress. Lastly, material parameter $\nu_{s}$ has a strong influence on the elastic limit strain and the compressive strength. Higher value of $\nu_{s}$ leads to earlier microcracking and failure due to faster lateral expansion. In contrast, a parametric study under uniaxial tension reveals that the (only) most sensitive parameter is $g_{f}$. The tensile strength of mortar increases with increasing $g_{f}$. Of all studied parameters, the initial aspect ratio or the initial microcrack radius appears to be highly influential on the compressive behaviour of the composites as compared to other parameters. It can be seen that the model is capable of simulating a wide range of stress–strain behaviour of cementitious materials.
